# Spatiotemporal Toxicity: Emerging Patterns of Industrial Water Pollution in the Coosa River Watershed, USA (2000–2020)

**DOI:** 10.1007/s00267-026-02457-6

**Published:** 2026-04-11

**Authors:** Al Artat Bin Ali, Jake Nelson

**Affiliations:** 1 Department of Geosciences, Auburn University, Auburn, AL USA; 2 Departement of Geography and Environment, University of Dhaka, Dhaka, Bangladesh

**Keywords:** Toxic Release Inventory (TRI), Industrial Pollution, Emerging Hotspot Analysis (EHSA), Spatial-Temporal Analysis, Water Contamination, Space-Time Cube Analysis

## Abstract

This study investigates the spatiotemporal dynamics of industrial toxic chemical discharges within the Coosa River watershed from 2000 to 2020 using the U.S. Environmental Protection Agency’s Toxics Release Inventory (TRI) dataset and advanced geospatial techniques. Findings reveal a contrasting trend in reported releases: while total on-site emissions to air, water, and land declined over time, reported surface-water discharges exhibited long-term growth and pronounced temporal variability. Nitrate and zinc compounds were among the most frequently reported chemicals released to surface waters. Emerging Hotspot Analysis (EHSA) and Space–Time Cube analysis identified persistent, intensifying, and newly emerging clusters of reported TRI surface-water discharge activity and facility concentration, particularly near long-established industrial centers around Gadsden. These hotspots reflect statistically significant spatial–temporal patterns in reported TRI discharges rather than measured in-stream contamination, exposure, or ecological impact. Despite overall reductions in reported emissions, the number and proportion of TRI facilities discharging to surface waters remained relatively stable, indicating persistent industrial discharge activity within the watershed. The findings demonstrate the value of TRI-based spatiotemporal analyses as screening-level tools for identifying areas of sustained or emerging industrial discharge pressure. Rather than indicating confirmed water quality impairment, the results provide a spatial framework to support future monitoring, data integration, and hypothesis-driven assessments in flood-prone river systems.

## Introduction

Industrial discharges have become a critical driver of water pollution, particularly in riverine environments where chemical inputs can threaten aquatic ecosystems and public health. Both natural and anthropogenic pressures on water resources create significant challenges for societal development and human well-being (Adikari et al., 2009; Ali et al., 2023). In recent decades, rapid industrial expansion, population growth, and shifting consumption patterns have intensified chemical release pressures on surface waters, contributing to widespread concerns about declining water quality (Alulema-Pullupaxi et al., 2021). The United Nations Educational, Scientific and Cultural Organization (UNESCO) has emphasized that deterioration in water quality undermines global water security and sustainable development, posing risks across both developed and developing contexts (UNESCO, 2015).

In the United States, rivers remain vital sources of freshwater for ecological, economic, and societal functions, yet they face mounting pressures from industrial effluents, untreated wastewater, and agricultural runoff. According to the U.S. Environmental Protection Agency’s (EPA) Toxics Release Inventory (TRI), industrial facilities reported nearly 200 million pounds of toxic chemical releases to surface waters in 2021, including substances classified as carcinogenic and those associated with reproductive and developmental health concerns (U.S. EPA, 2023). Major river systems such as the Mississippi, Ohio, and Missouri Rivers have been widely recognized as areas with elevated reported industrial discharge activity due to the concentration of manufacturing, energy production, and chemical processing facilities along their corridors (McLaughlin et al., 2020; König et al., 2017; Woodward et al., 2021). Importantly, these designations are based on reported industrial release data rather than comprehensive in-stream water quality measurements, highlighting the role of TRI as a screening-level indicator of potential industrial pressure on surface waters.

The persistence and spatial concentration of industrial discharges are of particular concern in river systems, where contaminants may be transported over long distances before dilution or attenuation occurs (McGauhey, 1968; Peavy, 1986). However, reported discharge quantities do not directly translate into observed contaminant concentrations, ecological effects, or human exposure, as these outcomes depend on hydrologic conditions, chemical fate and transport processes, and site-specific environmental characteristics. As a result, spatial analyses of TRI data are increasingly used to identify patterns of industrial activity and potential areas of concern rather than to diagnose confirmed water quality impairment.

This research focuses on the Coosa River watershed, a major river system in Alabama and northwestern Georgia that has experienced sustained industrial activity and long-term reporting of chemical releases under the TRI program (Chitwood, 2017). While prior studies have examined broad patterns of industrial pollution across large U.S. river basins, detailed spatiotemporal analyses that examine how reported TRI surface-water discharges evolve through space and time within the Coosa River watershed remain limited. Addressing this gap is particularly important given the watershed’s flood-prone character, which can influence the redistribution and spatial organization of reported industrial discharges.

To address this gap, the present study analyzes TRI-reported chemical releases from 2000 to 2020 using advanced geospatial and spatiotemporal techniques. This study examines patterns in reported TRI surface-water discharges and facility clustering to characterize how industrial discharge activity is organized and evolves across the watershed. The analysis integrates long-term TRI records with the Space–Time Cube framework and Emerging Hotspot Analysis (EHSA) to identify persistent, intensifying, and newly emerging clusters of reported discharge activity. By positioning hotspot patterns as indicators of relative reporting intensity rather than confirmed environmental impact, this approach provides a spatial screening tool that can help guide future monitoring, data integration, and hypothesis-driven research in the Coosa River watershed.

## Study Area

The Coosa River flows through large portions of Alabama and northwestern Georgia and is divided in this study into upper, middle, and lower reaches based on longitudinal position and watershed hydrology. The upper reach extends from the headwaters in northwestern Georgia to Weiss Lake, the middle reach spans from Weiss Lake to Lake Jordan, and the lower reach extends from Lake Jordan to the river’s confluence with the Alabama River. The river system has been subject to persistent pressures from both industrial effluents and diffuse agricultural runoff (Coosa Riverkeeper, n.d.). Reports by the Alabama Department of Environmental Management (ADEM) document water quality impairments within the basin, including regulatory listings on the 303(d) impaired waters list, which identifies surface waters that do not meet designated water quality standards under the U.S. Clean Water Act and require development of Total Maximum Daily Loads (TMDLs) (ADEM, 2024).

**Fig. 1:**
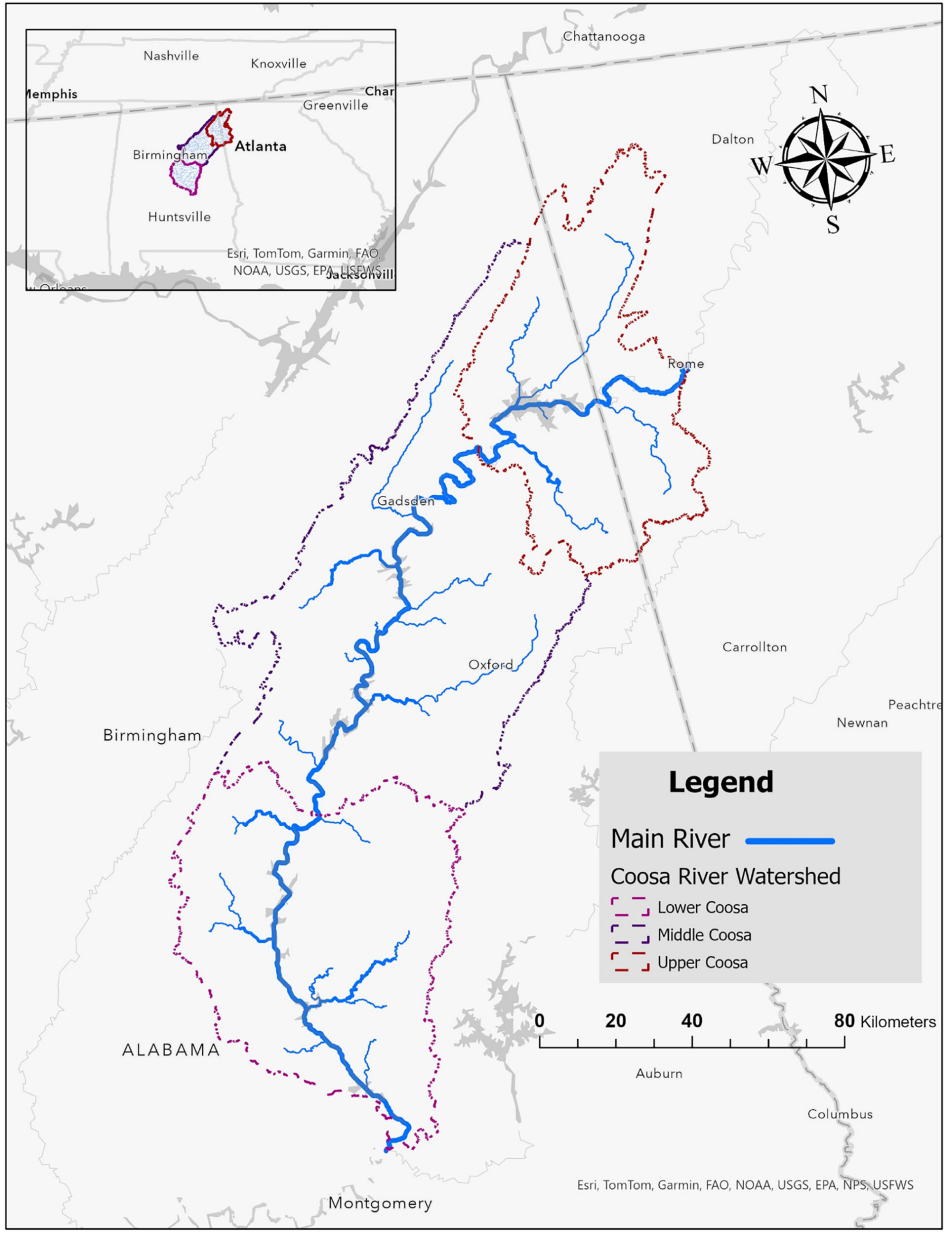
Geographic extent of the Coosa River watershed with HUC 8 delineated boundaries

Management plans for both the Coosa River Basin and the Lower Coosa River Basin identify industrial discharges as persistent stressors, particularly in river segments with high facility density and limited assimilative capacity (Coosa River Basin Management Plan, 1998; Lower Coosa River Basin Management Plan, 2005). Historical records further indicate sustained patterns of industrial discharge within the basin, raising concerns about cumulative contamination over time (Estis, 2008). Understanding the spatial distribution of TRI facilities across the upper, middle, and lower river reaches is therefore essential for evaluating longitudinal differences in discharge intensity and identifying areas of elevated water quality risk. The study area was selected based on the concentration of TRI facilities along the Coosa River mainstem and its major tributaries, where point-source discharges are most likely to influence surface water quality. Figure 1 presents the Coosa River watershed delineated using the Hydrologic Unit Code 8 (HUC-8) boundary and shows the spatial distribution of TRI facilities across the defined river reaches.

## Methods

### Toxic Release Inventory (TRI) Data

In this study, data from the U.S. Environmental Protection Agency’s (EPA) Toxic Release Inventory (TRI) are employed as the primary source of pollutant information (U.S. EPA, 2023). Facilities are included in the TRI if they manufacture, process, or otherwise handle designated quantities of toxic chemicals that exceed EPA thresholds for both chemical usage and workforce size. Such facilities are mandated to report annual releases of toxic substances to the EPA under the TRI program, which has systematically compiled and published discharge records since the enactment of the Emergency Planning and Community Right-to-Know Act (EPCRA) in 1986. The program was established to ensure public access to critical information regarding the release of hazardous chemicals from industrial and federal operations.

The TRI database provides comprehensive details on facility locations, quantities of chemical releases, pollutant categories, and the respective release pathways (air, water, and land). In this study, emphasis is placed on the total volume of chemicals reported by facilities located within the Coosa River watershed boundary. By examining TRI records from 2000 to 2020, shifts in the spatial distribution and intensity of toxic discharges were identified, revealing changes in contamination dynamics across the years 2000, 2010, and 2020. The dataset was therefore employed as the primary indicator of pollution sources, enabling the assessment of temporal patterns of toxic releases and their implications for contamination within the watershed.

### Spatial-Temporal Analysis

This study employed the Space Time Cube (STC) tool in ArcGIS Pro to investigate spatiotemporal trends in Toxic Release Inventory (TRI) water discharges within the Coosa River watershed between 2000 and 2020, using biennial intervals. The STC framework integrates spatial and temporal dimensions into discrete geographic units, enabling the detection of evolving pollution patterns over time (Rahaman et al., 2025). Each cube segment corresponds to a defined location and time step, with temporal trends evaluated through the Mann–Kendall test. This non-parametric test assesses monotonic changes by comparing all possible pairs of observations: an increase is assigned a score of +1, a decrease −1, and no change 0. Summing these scores provides an overall trend indicator, where a total of zero reflects no discernible trend. Statistical significance is subsequently evaluated using a z-score and associated p-value, with low p-values denoting significant trends and the sign of the z-score identifying whether the trend is positive or negative (ESRI, n.d.); Kendall and Gibbons, 1990; Mann, 1945).

The Mann–Kendall test statistic **S** is expressed as:

$$S=\,\mathop{\sum }\limits_{i=1}^{n-1}\mathop{\sum }\limits_{j=i+1}^{n}{a}_{{ij}}$$

where,

$${a}_{{ij}}={sign}\left({x}_{j}-{x}_{i}\right)={sign}\left({R}_{j}-{R}_{i}\right)=\left\{\begin{array}{c}1\\ 0\\ -1\end{array}\,\begin{array}{c}{x}_{i} < {x}_{j}\\ {x}_{i}={x}_{j}\\ {x}_{i} > {x}_{j}\end{array}\right.$$

Here, *R*_*i*_ and *R*_*j*_ represent the ranks of observations *x*_*i*_ and *x*_*j*_, respectively. Under the assumption that the data are independent and identically distributed, the expected value and variance of *S* are given as:

$$E\left(s\right)=0$$

$${V}_{o}\left(S\right)=\frac{n\left(n-1\right)\left(2n+5\right)}{18}$$

where *n* is the total number of observations. When tied ranks (equal observations) are present, the variance is adjusted as:

$${V}_{o}^{* }\left(S\right)=\frac{n\left(n-1\right)\left(2n+5\right)}{18}-\mathop{\sum }\limits_{j=1}^{m}\frac{{t}_{j}\left({t}_{j}-1\right)\left(2{t}_{j}+5\right)}{18}$$

Where *m* is the number of groups of tied ranks, each with *t*_*j*_ tied observations. The methods iterate over the independent data and calculate the exact distribution of *S*. The method makes the *S* closer to a normal distribution with the observation increment, which means the more the study time, the more accurate the model.

### Emerging Hotspot Analysis

The Space Time Cube (STC) framework was applied to detect temporal patterns in TRI water discharges by identifying spatial hotspots that emerged or persisted over time (ESRI, n.d.); Getis and Ord, 1992; Ord and Getis, 1995). This approach integrates the Getis–Ord local statistic, which builds on the variance reduction described in the following equation, and applies the following expression to compute the z-score (Ali et al., 2025):

$${G}_{i}^{* }=\frac{{\sum }_{j=1}^{n}{w}_{i,j}{x}_{j}-\bar{X}{\sum }_{j=1}^{n}{w}_{i,j}}{S\sqrt{\frac{\left[n{\sum }_{j=1}^{n}{w}_{i,j}^{2}-{\left({\sum }_{j=1}^{n}{w}_{i,j}\right)}^{2}\right]}{n-1}}}$$

where *x*_*j*_ is the attribute value for feature *j*, *w*_*i,j*_ represents the spatial weight between features *i* and *j*, and *n* denotes the number of features. The terms $\bar{X}$ and *S* are calculated using the following equations (Ali et al., 2026):

$$\bar{X}=\frac{{\sum }_{j=1}^{n}{x}_{j}}{n}$$

$$S=\sqrt{\frac{{\sum }_{j=1}^{n}{x}_{j}^{2}}{n}-{(\bar{X})}^{2}}$$

Using the Mann–Kendall statistics in conjunction with the STC framework, sixteen spatial–temporal cluster types were identified (ESRI, n.d.). These clusters were categorized according to their statistical significance and temporal evolution. A New Hot Spot emerges for the first time in the most recent time step, while a Consecutive Hot Spot appears in at least two sequential periods near the end of the series, but with less than 90% overall significance. An Intensifying Hot Spot remains significant in more than 90% of time steps with evidence of increasing intensity, whereas a Persistent Hot Spot also achieves significance in more than 90% of intervals but without notable changes in clustering strength. Conversely, a Diminishing Hot Spot reflects significant clustering that weakens across more than 90% of periods. A Sporadic Hot Spot appears intermittently, being significant only in the final step, while an Oscillating Hot Spot alternates between hot and cold status, with significance in fewer than 90% of steps. Finally, a Historical Hot Spot was significant in at least 90% of earlier intervals but lost significance in recent periods. Analogous categories were applied to cold spots (New, Consecutive, Intensifying, Persistent, Diminishing, Sporadic, Oscillating, and Historical), denoting clusters of low values under similar criteria. Together, this classification provides a nuanced understanding of spatial–temporal variability in contamination patterns within the Coosa River watershed, highlighting the importance of hotspot analysis in environmental research.

## Results

### Toxic Chemical Release Trends from TRI Facilities

A key factor influencing the extent to which contaminated waters affect nearby communities is the volume of pollutants discharged from TRI facilities. Accordingly, this study examined the total onsite release of toxic chemicals to air, water, and land from 2000 to 2020 within the Coosa River watershed to evaluate long-term discharge patterns.

**Fig. 2:**
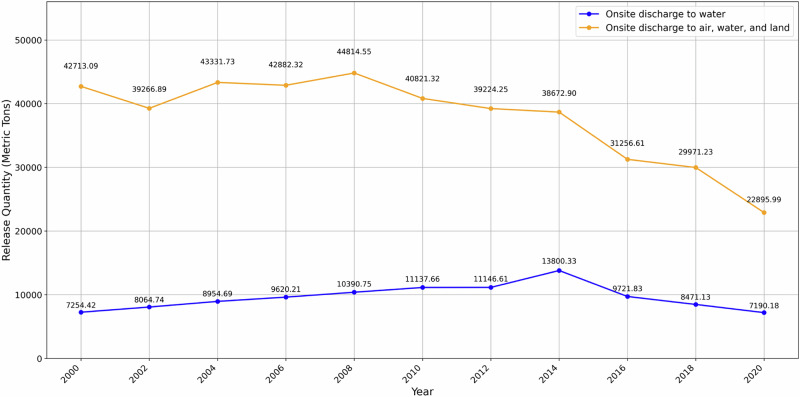
Trend in total chemical releases (metric tons) from Toxic Release Inventory facilities across 2000–2020 at two-year intervals

Figure 2 presents temporal trends in onsite chemical releases, distinguishing surface water discharges (blue line) from the combined releases to air, water, and land (orange line), expressed in metric tons. Surface water discharges increased from ~7250 metric tons in 2000 to a maximum of 13,800 metric tons in 2014, before declining to about 7200 metric tons by 2020. In contrast, total onsite releases across all media declined over the study period despite early variability. Combined releases were ~42,700 metric tons in 2000, increased to a peak of about 44,800 metric tons in 2008, and then decreased steadily to roughly 22,900 metric tons in 2020.

**Fig. 3:**
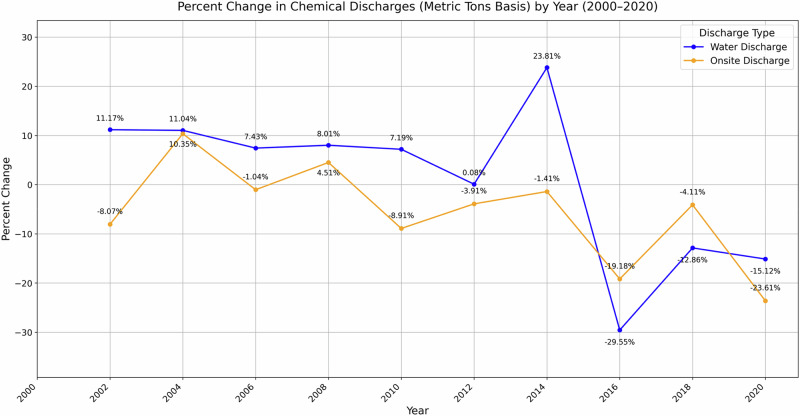
Percent change in chemical releases reported by TRI facilities at two-year intervals between 2000 and 2020

Figure 3 illustrates the percent change in onsite chemical discharges at two-year intervals, distinguishing surface water releases from combined onsite discharges to air, water, and land. Surface water discharges show pronounced temporal variability, increasing by ~11% in 2002 and 2004, followed by moderate gains through 2010, and a sharp rise of about 24% in 2014. This increase was followed by a substantial reversal, with a marked decline of 53.36% between 2014 and 2016 (from +23.81% to −29.55%), after which surface water discharges remained negative through 2020. In contrast, combined onsite discharges exhibit relatively smaller fluctuations, increasing by about 10% in 2004 and 5% in 2008, declining by approximately 9% in 2010, and experiencing a notable reduction of about 18% between 2014 and 2016 (from −1.41% to −19.18%), followed by further decreases by 2020. Overall, these contrasting trends indicate that while total onsite emissions have generally declined over time, surface water discharges have been characterized by sharper and more volatile shifts.

#### Chemical Emissions

**Fig. 4:**
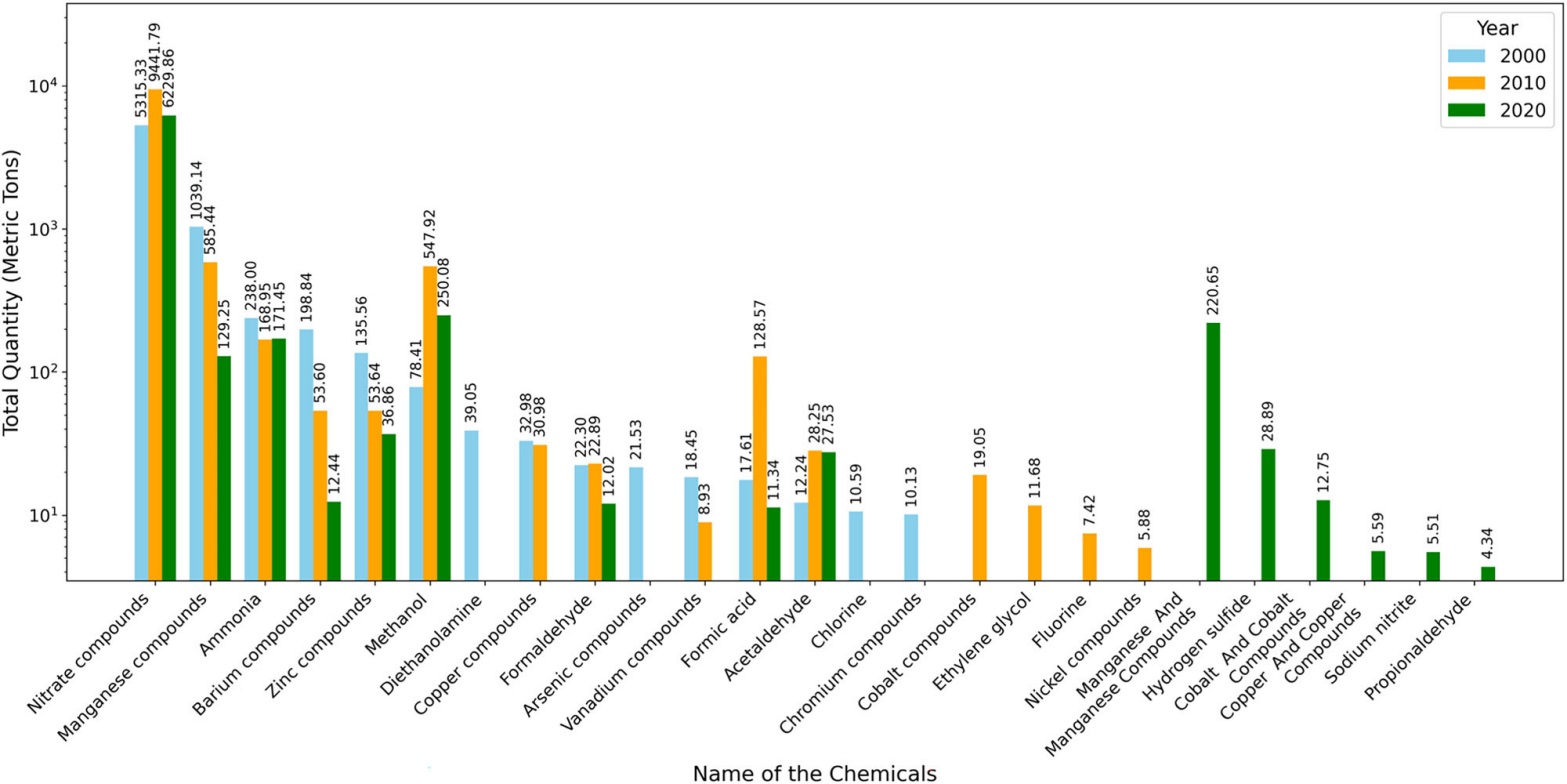
Top 15 chemical releases (total quantity in log scale) from TRI facilities during 2000, 2010, and 2020

Figure 4 illustrates the surface water discharges of the top 15 chemicals in 2000, 2010, and 2020, expressed in metric tons. Although a total of 56 distinct chemicals were reported across the three study years, the analysis focuses on the top contributors based on release magnitude to capture the chemicals accounting for the majority of surface water discharges. This approach facilitates a clear comparison of temporal changes in both the composition and relative magnitude of the most dominant pollutants.

In 2000, nitrate compounds dominated surface water discharges, with releases of approximately 5300 metric tons, followed by manganese compounds at about 1040 metric tons. Ammonia and zinc compounds were released at moderate levels, each on the order of 200 metric tons, while barium compounds contributed roughly 200 metric tons. Additional chemicals, including methanol (about 80 metric tons), diethanolamine (about 40 metric tons), copper compounds (about 33 metric tons), formaldehyde (about 22 metric tons), and arsenic compounds (about 22 metric tons), were present at comparatively lower levels.

By 2010, nitrate compounds remained the most prevalent contaminant, increasing to approximately 9400 metric tons, while manganese compounds declined to about 585 metric tons. Methanol increased substantially to nearly 550 metric tons, emerging as one of the major contributors. Zinc compounds decreased to around 54 metric tons, and barium compounds declined to ~130 metric tons. Several additional chemicals appeared among the top discharges, including formic acid (about 130 metric tons), acetaldehyde (about 28 metric tons), cobalt compounds (about 19 metric tons), ethylene glycol (about 12 metric tons), and fluorine (about 7 metric tons).

In 2020, nitrate compounds continued to dominate surface water releases, totaling approximately 6200 metric tons. Methanol remained elevated at about 250 metric tons, while manganese compounds declined further to roughly 130 metric tons. Manganese associated with manganese compounds emerged prominently, with releases of ~220 metric tons, along with hydrogen sulfide (about 30 metric tons) and cobalt and cobalt compounds (about 13 metric tons). Several additional substances, including sodium nitrite, propionaldehyde, and copper compounds, were reported at levels below 10 metric tons.

Overall, the results indicate persistent dominance of nitrate compounds in surface water discharges across all three years, alongside substantial shifts in the relative contributions of other chemicals. While some compounds exhibited declining release quantities over time, others emerged or increased in later years, reflecting changes in the composition of surface water discharges within the watershed.

### Emission Industries

**Fig. 5:**
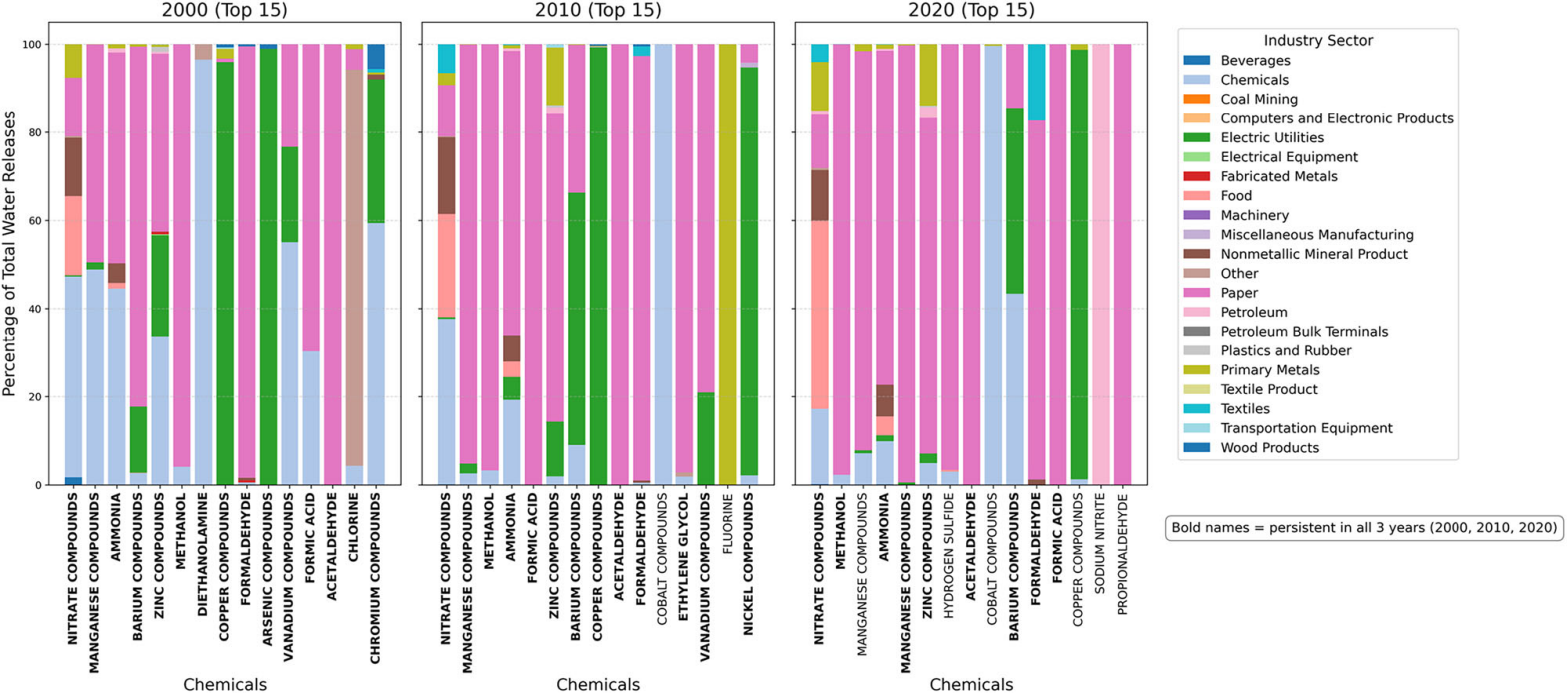
Distribution of industry sectors (TRI facilities) contributing to the release of the top 15 chemicals in 2000, 2010 and 2020

In addition to reporting releases by chemical type and total quantity, the TRI dataset attributes surface water discharges to specific industrial sectors. Figure 5 depicts the industry-sector composition of surface water releases for the top 15 chemicals in 2000, 2010, and 2020, shown as stacked bars where each color represents the percentage contribution of an industry sector to the total release of a given chemical. Each panel corresponds to a study year, enabling visual comparison of sectoral contributions both within individual chemicals and across time.

Across all three years, releases of nitrate compounds, ammonia, and manganese compounds (shown in bold) persist among the top contributors, indicating their continued prominence in surface water discharges over time. For many chemicals, the paper manufacturing sector constitutes the dominant source, particularly for nitrate compounds and several organic chemicals, accounting for the majority of releases in each year. The electric utilities sector also emerges as a major contributor, especially for metals and metal-related compounds, while the chemical manufacturing sector contributes substantially to selected compounds such as methanol and formaldehyde. Although the relative sectoral shares vary by chemical and year, Fig. 5 highlights the consistent dominance of a small number of industrial sectors and underscores the persistence of specific chemicals across all three study periods.

### Distribution of TRI Facilities in the Coosa River Watershed

Table 1 summarizes the number of TRI facilities within the watershed from 2000 to 2020, distinguishing facilities releasing ≥100 pounds (0.05 tons) of chemicals to air, water, and land from those discharging directly to surface waters. In 2000, a total of 184 facilities reported releases across all media. This number fluctuated over the study period, reaching a maximum of 192 facilities in 2006 before declining to 160 facilities by 2020. Facilities reporting direct surface water discharges exhibited a similar pattern, increasing from 37 facilities in 2000 to a peak of 41 facilities in 2010, followed by a decline to 33 facilities in 2020. The proportion of facilities contributing to surface water discharges varied over time, decreasing from 20.10% in 2000 to 14.67% in 2002, increasing gradually thereafter, and stabilizing at 20.62% in 2020.

**Table 1:** Total number of TRI facilities located in the Coosa River watershed over the period 2000–2020

Year	Number of TRI facilities releasing ≥ 100 pounds (0.05 tons) of chemicals to land, water, and air.	Number of TRI facilities releasing ≥ 100 pounds (0.05 tons) of chemicals to surface water.	Percentage of TRI facilities releasing chemicals to surface water.
2000	184	37	20.10%
2002	184	27	14.67%
2004	191	37	19.37%
2006	192	35	18.22%
2008	182	37	20.32%
2010	185	41	22.16%
2012	168	37	22.02%
2014	180	35	19.44%
2016	165	32	19.39%
2018	169	34	20.11%
2020	160	33	20.62%

### Emerging Hotspot Analysis of the TRI Facilities (from 2000 to 2020)

The Emerging Hotspot Analysis (EHSA) conducted for the Coosa River watershed over the period 2000–2020 provides a detailed picture of how the spatial and temporal patterns of reported TRI surface-water discharges and facility clustering have evolved. It is important to emphasize that the identified hotspots represent statistically significant spatial–temporal concentrations of reported TRI discharge activity, rather than direct measurements of water quality, contaminant concentrations, or human or ecological exposure. This approach highlights not only areas of persistent discharge activity but also locations where new reporting patterns have recently emerged. Together, the results help identify regions that may warrant closer regulatory attention and targeted management review, without implying confirmed water quality impairment in the absence of independent monitoring data.

**Fig. 6:**
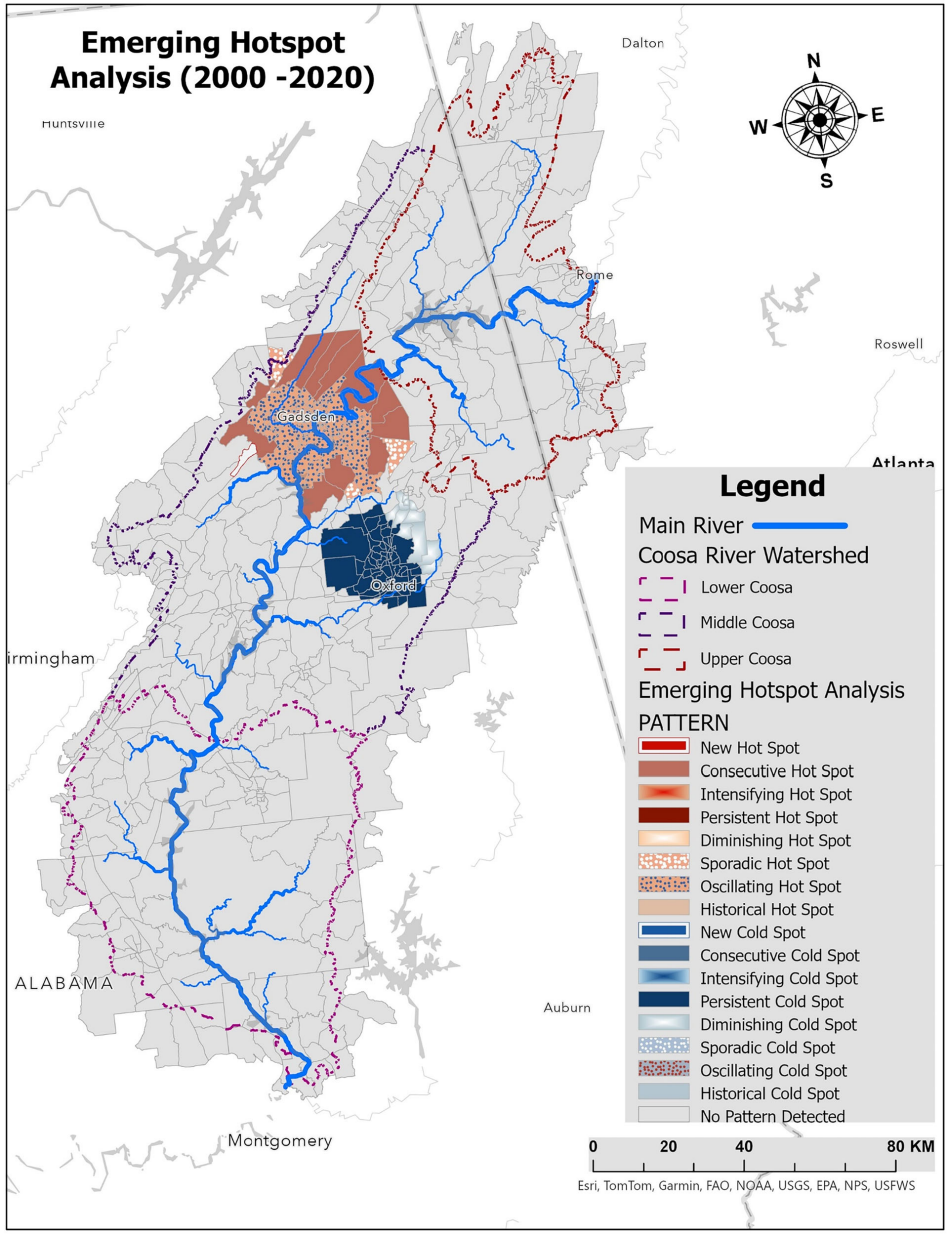
Map of Emerging Hotspot Analysis outcomes for TRI facilities (2000–2020), revealing patterns

As illustrated in Fig. 6 and Table 2, consecutive hotspots occupy 3.27% of the watershed. These hotspots indicate locations where reported TRI surface-water discharges and facility activity have remained consistently elevated across multiple time periods, reflecting sustained industrial influence in terms of reporting intensity rather than measured contamination levels. Such hotspots are predominantly concentrated in the upper and middle sections of the watershed, particularly surrounding the city of Gadsden, an area historically associated with dense industrial activity. By contrast, diminishing cold spots account for 0.63% of the watershed and represent areas where reported TRI discharges have declined over time, suggesting reduced reporting intensity or changes in industrial discharge practices. New hotspots, although covering only 0.07% of the watershed, are particularly noteworthy because they signal emerging clusters of reported TRI discharge activity, where surface-water releases have recently increased relative to prior periods. The analysis also identifies sporadic hotspots, representing 0.42% of the watershed. These zones reflect intermittent clustering of reported TRI discharges, characterized by irregular episodes of elevated activity rather than sustained trends, which may be associated with episodic industrial operations or reporting variability. A striking observation is that the majority of the watershed, covering approximately 90.4% of its area (indicated in grey), shows no statistically significant spatial–temporal clustering of TRI surface-water discharges. These “no trend” areas should not be interpreted as contamination-free or low-risk zones, but rather as locations where reported discharge activity does not exhibit significant clustering at the spatial and temporal scales evaluated.

**Table 2:** Patterns identified by Emerging Hotspot Analysis (2000–2020) and proportion of area in each category

Patterns observed	Percentage of area
Consecutive Hot Spot	3.27%
Diminishing Cold Spot	0.63%
New Hot Spot	0.07%
No Pattern Detected	90.4%
Oscillating Hot Spot	2.82%
Persistent Cold Spot	2.28%
Sporadic Hot Spot	0.42%

In the middle Coosa region near Oxford, more complex spatial–temporal patterns were observed. Oscillating hotspots, covering 2.82% of the watershed and depicted in orange, indicate areas where reported TRI discharges fluctuated between higher and lower levels across time intervals, reflecting variable industrial activity or discharge practices. Persistent cold spots, which make up 2.28% of the watershed and are shown in dark blue, represent areas with consistently low levels of reported TRI surface-water discharges, suggesting limited industrial reporting intensity rather than confirmed absence of contamination pressure.

## Discussion

This study evaluated the spatial and temporal dynamics of Toxic Release Inventory facilities and their surface water discharges within the Coosa River watershed, with a focus on pollution behavior in a flood-prone river system. The results demonstrate a clear decoupling between overall emission reductions and surface water discharge patterns. As shown in Figs. 2 and 3, total on-site releases across air, water, and land declined over time, while surface water discharges followed a contrasting trajectory characterized by long-term growth and pronounced variability. Importantly, these trends represent changes in reported TRI surface water discharges rather than direct measurements of in-stream water quality or contaminant concentrations. This distinction is critical because TRI data reflect facility-reported release quantities and locations, not observed environmental conditions or exposure outcomes.

The contrasting trends observed in Fig. 2 underscore the importance of pathway-specific interpretation of TRI data. While combined releases declined steadily after the mid-2000s, surface water discharges increased during the same period and exhibited sharper inter-annual fluctuations, as highlighted in Fig. 3. These patterns indicate shifts in the magnitude and spatial organization of reported industrial discharges. Similar divergences have been reported in previous TRI-based studies, which show that regulatory controls and technological advances often prioritize air emission reductions, whereas waterborne discharges remain more sensitive to facility-level operational practices and wastewater management strategies (Wilson et al., 2012). Within flood-prone watersheds, such pathway-specific trends may be further amplified by hydrologic variability, increasing the relevance of surface water reporting for screening-level risk assessments. In the Coosa River watershed, this imbalance suggests that reliance on aggregate emissions alone may obscure evolving discharge patterns relevant to surface water management.

Chemical-specific results further clarify the nature of these discharge patterns. Figure 4 shows that nitrate compounds consistently dominated reported surface water discharges across all benchmark years. This persistence reflects the continued prominence of nutrient-related industrial releases in TRI reporting. Nutrient-related discharges are widely recognized as potential long-term stressors in riverine environments due to their interaction with hydrologic variability and flood-driven transport processes (Liévanos, 2018). Previous flood–contamination studies in the Coosa River basin similarly highlight nutrients as recurring contributors to compound risk patterns, particularly where industrial activity intersects with flood-prone landscapes (Ali and Nelson, 2025). However, the results here should be interpreted as indicating sustained discharge pressure rather than verified ecological impact.

Shifts in the composition and ranking of other chemicals shown in Fig. 4 reflect evolving industrial practices rather than uniform emission reductions. The emergence and re-ranking of organic compounds and metals over time are consistent with TRI-based spatial analyses that attribute such changes to modifications in production processes, raw material inputs, and wastewater treatment strategies (Hou et al., 2022). These compositional changes highlight variability in reported discharge profiles, not measured changes in environmental concentrations, underscoring the importance of adaptive monitoring frameworks.

Sector-level results presented in Fig. 5 provide additional context for understanding the persistence of reported surface water discharges. The dominance of a limited number of industrial sectors across all three study years indicates that discharge activity is driven by concentrated industrial sources rather than diffuse contributions. This finding aligns with prior TRI research showing that a small subset of sectors accounts for a disproportionate share of reported releases (Ding et al., 2015). From a management perspective, this sectoral concentration identifies where discharge reporting and oversight efforts may be most effectively focused, without implying sector-specific environmental damage.

Facility-level trends summarized in Table 1 further reinforce this interpretation. Although the total number of TRI facilities declined over time, the proportion of facilities reporting surface water discharges remained relatively stable. This pattern indicates persistence in the spatial distribution of discharge-reporting facilities rather than changes in observed water quality conditions. Similar persistence has been noted in prior spatiotemporal analyses of industrial risk in the Coosa River watershed, where reductions in facility counts did not translate directly into proportional reductions in contamination potential (Ali and Nelson, 2025). As a result, reductions in facility counts alone should not be interpreted as proportional reductions in surface water contamination or exposure risk.

The spatiotemporal structure revealed through Emerging Hotspot Analysis provides a spatial framework for integrating these findings. As illustrated in Fig. 6 and summarized in Table 2, consecutive and sporadic hotspots are concentrated near long-established industrial centers, particularly in the upper and middle reaches of the watershed. In this study, hotspots represent statistically significant clustering of reported TRI surface water discharges and facility locations through time, rather than confirmed contamination hotspots or areas of measured water quality impairment. Similar spatial-temporal analyses have shown that industrial corridors often function as long-term anchors of reported discharge activity within river systems (Ding et al., 2015).

Newly emerging hotspots, though limited in spatial extent, are particularly important because they indicate areas where reported discharge activity is increasing or becoming more spatially clustered, rather than areas where environmental impacts have been confirmed. Conversely, areas classified as having no statistically detectable pattern should not be interpreted as risk-free zones. Such classifications reflect spatial stability or diffuse reporting patterns at the scale of analysis, not the absence of contamination or exposure. Hotspot analyses, therefore, complement, rather than replace, direct water quality monitoring and exposure assessment.

By linking pathway-specific discharge trends with chemical composition, sectoral dominance, facility persistence, and spatiotemporal clustering, this study demonstrates that TRI-based hotspot patterns capture the organization and evolution of reported industrial discharges rather than confirmed environmental conditions. The findings emphasize the value of spatially explicit TRI analyses for identifying persistent and emerging patterns of industrial activity that may warrant further investigation. For flood-prone watersheds such as the Coosa River, integrating TRI-based spatiotemporal insights with hydrologic and water quality data can help guide targeted monitoring, regulatory prioritization, and future risk assessment efforts without overstating environmental impacts.

## Concluding Remarks

This study delivers a comprehensive evaluation of the spatiotemporal dynamics of TRI releases in the Coosa River watershed, offering new insights into industrial pollution patterns across two decades. By applying Emerging Hotspot Analysis alongside the Space–Time Cube framework, persistent, intensifying, and newly emerging clusters of industrial discharge activity were systematically identified, addressing an important gap in understanding how reported surface-water releases vary through space and time in this region. The results highlight a contrasting trend: although total on-site emissions to air, water, and land have declined overall, surface water discharges have increased, particularly for nitrate and zinc compounds, suggesting a shift in the dominant pathway of reported industrial releases rather than direct evidence of worsening in-stream conditions. Accordingly, the identified hotspot patterns should be interpreted as screening-level indicators of potential concern, instead of direct measures of ecological harm or regulatory noncompliance.

Several important data and methodological limitations should be acknowledged to ensure transparency and appropriate interpretation of the findings. First, this analysis relies exclusively on TRI-reported release estimates and does not incorporate in situ water quality measurements or National Pollutant Discharge Elimination System compliance data, which limits the ability to verify reported releases against observed concentrations or regulatory thresholds. Second, the study does not assess contaminant fate, transport, dilution, or exposure pathways within the river system, and therefore cannot directly evaluate downstream concentrations, ecological impacts, or human exposure risks. Third, TRI data are subject to potential reporting biases, including underreporting, estimation uncertainty, reporting exemptions, and facility-level inconsistencies, which may influence both temporal trends and spatial clustering patterns. These limitations indicate that the results are best interpreted as a spatial characterization of reported industrial discharge activity rather than a definitive assessment of water quality impairment.

The management and policy implications derived from this study should therefore be viewed as hypothesis-generating rather than prescriptive. While the concentration of consecutive and sporadic hotspots within established industrial centers highlights areas where closer monitoring or further investigation may be warranted, these patterns do not by themselves indicate regulatory failure or confirmed environmental degradation. Instead, they provide a spatial framework for prioritizing future data collection, monitoring, and process-based studies.

Future research should integrate TRI data with water quality monitoring, NPDES discharge records, and hydrological modeling to better evaluate contaminant transport, dilution, and persistence within the watershed. Linking reported releases to observed chemical concentrations, biological indicators, and sediment dynamics would allow for a more robust assessment of ecological and human health relevance. Additionally, incorporating climate variability and extreme hydrologic events into future analyses could clarify how flooding influences pollutant redistribution and exposure potential. By positioning TRI-based hotspot analyses as an initial screening tool that guides subsequent targeted investigations, future work can more effectively support evidence-based environmental management and sustainable water resource planning in the Coosa River watershed and similar flood-prone systems.

## Data Availability

No datasets were generated or analysed during the current study.
